# Mitochondrial DNA Copy Number in Peripheral Blood Is Independently Associated with Visceral Fat Accumulation in Healthy Young Adults

**DOI:** 10.1155/2014/586017

**Published:** 2014-02-24

**Authors:** Jee-Yon Lee, Duk-Chul Lee, Jee-Aee Im, Ji-Won Lee

**Affiliations:** ^1^Department of Family Medicine, Severance Hospital, Yonsei University, College of Medicine, 250 Seongsanno, Seodaemun-gu 120-752, Republic of Korea; ^2^Sport and Medicine Research Center, INTOTO Inc., 401 Dawoo BD, 90-6 Daeshin-Dong, Seodaemun-gu, Seoul 120-160, Republic of Korea

## Abstract

*Aims*. Visceral obesity is associated with an increased risk of cardiometabolic diseases and it is important to identify the underlying mechanisms. There is growing evidence that mitochondrial dysfunction is associated with metabolic disturbances related to visceral obesity. In addition, maintaining mitochondrial DNA (mtDNA) copy number is important for preserving mitochondrial function. Therefore, we investigated the relationship between mtDNA copy number and visceral fat in healthy young adults. *Methods*. A total of 94 healthy young subjects were studied. Biomarkers of metabolic risk factors were assessed along with body composition by computed tomography. mtDNA copy number was measured in peripheral leukocytes using real-time polymerase chain reaction (PCR) methods. *Results*. The mtDNA copy number correlated with BMI (*r* = −0.22, *P* = 0.04), waist circumference (*r* = −0.23, *P* = 0.03), visceral fat area (*r* = −0.28, *P* = -0.01), HDL-cholesterol levels (*r* = 0.25, *P* = 0.02), and hs-CRP (*r* = 0.32, *P* = 0.02) after adjusting for age and sex. Both stepwise and nonstepwise multiple regression analyses confirmed that visceral fat area was independently associated with mtDNA copy number (*β* = -0.33, *P* < 0.01, *β* = 0.32, and *P* = 0.03, resp.). *Conclusions*. An independent association between mtDNA content and visceral adiposity was identified. These data suggest that mtDNA copy number is a potential predictive marker for metabolic disturbances. Further studies are required to understand the causality and clinical significance of our findings.

## 1. Introduction

The prevalence of obesity is increasing worldwide. Obesity is a well-known risk factor for numerous health problems, including cardiovascular disease (CVD), diabetes mellitus (DM), and cancer [1, 2]. Recent data shows that the regional distribution of body fat, rather than overall obesity, contributes to disease processes [3]. Visceral fat is more metabolically active than subcutaneous fat [4] and affects the development of metabolic disturbances, including insulin resistance [5] and dyslipidemia [6]. The precise role of visceral adiposity in metabolic disturbance is still unknown but proinflammatory cytokines and adipokines secreted by visceral adipocytes are believed to be involved [7].

Mitochondria are organelles that play an important role in the energy synthesis of the cells. Mitochondria synthesize the molecules essential for the body metabolism and influence metabolic homeostasis of the entire body [8]. Mitochondrial function decreases with aging and mitochondrial dysfunction is related to various age-related conditions including type 2 DM and CVD [9]. Mitochondria are highly vulnerable to oxidative damage [10, 11] and mitochondrial dysfunction induced by oxidative damage is considered to contribute to the development of cardiometabolic diseases [9].

Mitochondrial DNA copy number, which reflects the content of mtDNA, is associated with mitochondrial gene stability and mitochondrial biogenesis [12]. Mitochondrial dysfunction reduces the contents of mitochondria, which is expressed as a decreased mtDNA copy number [12]. Furthermore, reduced mitochondrial DNA content of peripheral blood as well as specific organs was associated with the development of IR, type 2 DM [13], cognitive function [14], and metabolic syndrome [15]. Adipose tissue is the main source of cytokines and adipokines that increase systemic oxidative stress. Thus, obesity may decrease mitochondrial function. Previous results show that human obesity is associated with mitochondrial dysfunction. However, few studies have investigated the quantitative changes in mitochondria according to increased adiposity. The studies that have been performed have yielded mixed results. Furthermore, the potential differences in mitochondrial content according to the regional distribution of adiposity were not fully evaluated.

Adipose tissue is the main source of cytokines and adipokines that increase systemic oxidative stress [16]. Thus, obesity may decrease mitochondrial function. Previous results show that human obesity is associated with mitochondrial dysfunction [17]. However, few studies have investigated the quantitative changes in mitochondria according to increased adiposity. The studies that have been performed have yielded mixed results [18, 19]. Furthermore, the potential differences in mitochondrial content according to the regional distribution of adiposity were not fully evaluated.

Therefore, we investigated the association between peripheral blood mtDNA copy number and visceral fat accumulation among 94 healthy young-aged people.

## 2. Materials and Methods

### 2.1. Study Sample

This was a secondary data analysis from the Yonsei Aging Cohort, which was designed to investigate health-related markers among people of various ages [20]. Participants visited the Department of Family Medicine at Severance Hospital for routine health checkups and not for investigations or treatments of specific symptoms or diseases. All subjects participated in the study voluntarily, and written informed consent was obtained from each participant. Questionnaires about lifestyles and underlying medical conditions, overnight-fasting blood tests, and fat measurements with computed tomography were performed as baseline tests. Two additional samples of blood were collected from participants who agreed to store their blood samples for 10 years for future analysis. An additional separate written informed consent was obtained from each participant before performing the additional laboratory test with the stored blood samples.

Because the aim of our study was to investigate the association between visceral obesity and mtDNA copy number in healthy young participants, we selected 203 people aged from 20 to 40 years. Mitochondrial DNA copy numbers were measured with the stored blood samples. Thus we excluded 75 participants who did not agree to store their blood samples. Fifteen additional participants were excluded, because data for their abdominal visceral fat areas were missing. To select a healthy population, participants with histories of hypertension, diabetes mellitus, coronary artery occlusive disease, chronic liver disease, chronic renal disease, or cancer were not included. Subjects who participated in regular exercise were also excluded from the data analysis. Regular exercise was defined as physical exercise or physical work that was performed for more than 30 minutes, three times per week. We also excluded participants who used medications, including antihypertensive agents, lipid-reducing drugs, oral hypoglycemic agents, and nutrient supplements, which could affect cardiometabolic functions. Ninety-four patients were included in our analyses. The study complied with the Declaration of Helsinki, and the institutional review board of Yonsei University College of Medicine approved this study.

### 2.2. Measurements

All participants were questioned about lifestyle factors, including alcohol consumption and smoking. Alcohol consumption was defined as drinking alcohol more frequently than once per week. Smoking was defined as current cigarette smoking.

Anthropometric measurements were made by a single examiner. After a 10-minute resting period, blood pressure was measured in the sitting position. Body mass index was calculated as weight (kg) divided by height squared (cm^2^).

Abdominal fat tissue area was calculated using computed tomography (Tomoscan 350; Philips, Mahwah, NJ, USA) as described previously [21].

Blood samples were collected after at least an 8-hour overnight fasting period. Fasting glucose, high sensitive C-reactive protein (hs-CRP), total cholesterol, triglyceride, and high-density lipoprotein (HDL) cholesterol levels were measured using an ADVIA 1650 chemistry system (Siemens Medical Solution, Tarrytown, NY, USA). Fasting insulin levels were determined using electrochemiluminescence immunoassays with an Elecsys 2010 (Roche, Indianapolis, IN, USA). Insulin resistance was calculated using the homeostasis model assessment of insulin resistance (HOMA-IR) index: (insulin [*μ*IU/mL] × fasting blood glucose [mg/dL]/18)/22.5.

### 2.3. Measurement of Mitochondrial DNA Copy Numbers in Peripheral Blood

To reduce variations in measurements, one examiner measured all parameters throughout the study. mtDNA in peripheral leukocytes was extracted from 1 mL of whole blood using the QIAamp Tissue Kit 250 (Qiagen Inc., Valencia, CA, USA). The relative mtDNA copy number was measured by a real-time polymerase chain reaction (QPCR) and corrected by simultaneous measurement of the nuclear DNA according to the method of Wong and Cortopassi [22] and Liu et al. [11]. Reactions were performed using a Light Cycler-Fast Start DNA Master SYBR Green I kit, purchased from Roche Applied Science (Pleasanton, CA, USA). The forward and reverse primers of *β*-globin (used to amplify a 268 bp product) were 5′-GAAGAGCCAAGGACAGGTAC-3′ and 5′-CAACTTCATCCACGTTCACC-3′, respectively. The forward and reverse primers of the mitochondrial gene (ND1 gene) used to amplify a 153 bp product were 5′-AACATACCCATGGCCAACCT-3′ and 5′-AGCGAAGGGTTGTAGTAGCCC-3′, respectively. After denaturation at 95°C for 300 seconds, DNA samples were treated at 95°C for 0.1 seconds, 58°C for 6 seconds, and 72°C for 18 seconds for 40 cycles. A total of 20 ng of DNA was used and the number of PCR cycles to reach this amount of DNA was defined as the threshold cycle (Ct). The following equation was used to quantify the mtDNA copy number relative to *β*-globin: relative copy number = 2^ΔCt^ (ΔCt = Ct_*β*-globin_ − Ct_ND1_) [23]. The intra-assay and interassay coefficients of variation of Ct values for the ND1 gene were 4.5% and 5.8%, respectively.

### 2.4. Statistical Analyses

Normally distributed data are expressed as the mean ± standard deviation (SD). Nonnormally distributed data are expressed as median and interquartile range. mtDNA, fasting insulin, HOMA-IR, total cholesterol, triglyceride, and hs-CRP were log transformed to improve the skewness of the distribution. Pearson correlation analyses were performed to evaluate relationships between mtDNA and other metabolic variables. Stepwise multiple linear regression analysis was performed to identify factors that contributed to mtDNA copy number. If there was a significant correlation (*r* > 0.7) between two variables, only one variable was selected and entered into the model to avoid multicollinearity. In addition, nonstepwise multiple linear regression analysis was performed. Variables with *P* < 0.05 in the univariate analysis and clinically important variables, including age, BMI, and HOMA-IR, were entered into the nonstepwise analysis.

We performed all statistical analyses using the Statistical Package for the Social Sciences, version 18.0 (SPSS Inc., Chicago, IL, USA). Statistical significance was defined as *P* < 0.05.

## 3. Results

**Figure 1:**
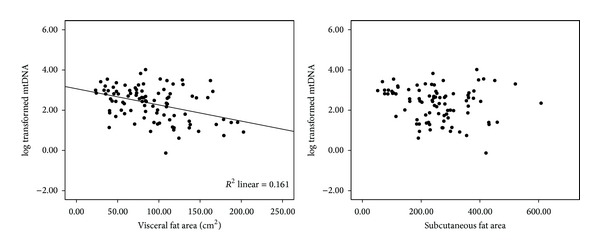
The relationship between abdominal visceral fat area, abdominal subcutaneous fat area, and mtDNA copy numbers. Coefficients (*r*) and *P* values were calculated using the Pearson correlation model.

The clinical characteristics of the study subjects are shown in Table 1. The mean age of the study subjects was 32.26 ± 9.14 years, and the median (25–75th percentile) mtDNA copy number was 302.08 (48.98–891.25). After adjusting for age and sex, mtDNA copy numbers positively correlated with HDL-cholesterol levels (*r* = 0.25, *P* = 0.02) and negatively correlated with BMI (*r* = −0.22, *P* = 0.04), waist circumference (*r* = −0.23, *P* = 0.03), visceral fat area (*r* = −0.28, *P* = −0.01), and hs-CRP (*r* = 0.32, *P* = 0.02) (Table 2). The mean mtDNA level in the nonsmoking group (2.50 ± 0.81) was significantly higher than that of the smoking group (1.80 ± 0.74, *P* < 0.001). In addition, the mean mtDNA level of female subjects (2.57 ± 0.80) was significantly higher than that of male subjects (2.15 ± 0.83, *P* = 0.02). There were no significant differences in mean mtDNA levels between subjects that consumed alcohol (2.17 ± 0.76) and subjects that did not (2.42 ± 0.88, *P* = 0.17).  Figure 1 shows the different relationships between mtDNA copy number and abdominal adiposity according to the regional fat distribution. The mtDNA copy numbers negatively correlated with visceral fat area. However, there was no significant correlation with subcutaneous fat.

In stepwise multiple linear regression analyses, visceral fat area, hs-CRP, HDL-cholesterol, and smoking accounted for 35% of the variance in mtDNA copy number. Thus, these variables were considered to be explanatory variables for mtDNA copy number. In addition, nonstepwise multiple regression analyses indicated that visceral fat, smoking, and hs-CRP levels were independently associated with mtDNA copy numbers, as these variables accounted for 58% of the variance (Table 3).

## 4. Discussion

Our cross-sectional study revealed a relationship between peripheral blood mtDNA copy number and visceral obesity in a healthy Korean young-aged population. This association remained significant after adjusting for BMI and other confounding factors. In addition, our study showed a significant relationship between mtDNA copy number with smoking, the components of metabolic syndrome (waist circumference, blood pressure, and HDL-cholesterol), and cardiovascular risk factors (systolic and diastolic BP), which is consistent with the findings of previous studies [15, 24].

The mitochondrion is an organelle with diverse functions, including energy synthesis, cellular remodeling, and regulation of cell metabolism. Mitochondrial dysfunction induces various metabolic diseases, including insulin resistance, type-2 diabetes mellitus, and CVD [9]. Multiple biochemical mechanisms, including impaired fatty-acid oxidation, and mitochondrial reactive oxygen stress, explain the link between mitochondrial dysfunction and pathologic conditions [25]. Although mitochondria are present in all types of cells, increasing evidence indicates that mitochondrial function in adipocytes is important for metabolic regulation. In an experimental animal model, rats with visceral obesity showed defective oxidative metabolism and reduced mitochondrial gene expression [26]. Kraunsøe et al. reported that mitochondrial respiration was reduced in the visceral adipose tissues of obese humans compared to that in subcutaneous fat tissues [27]. Furthermore, obese people have been shown to have defective mitochondrial ATP formation compared with that of nonobese people [28]. However, there are mixed results from human clinical studies regarding the quantitative aspects of mtDNA and regional distribution of adiposity. Yin et al. reported a modest decrease in mtDNA content of omental adipocytes from obese men compared with that of nonobese men [29]. In a Korean study, an inverse relationship was reported between peripheral mitochondrial DNA copy number and visceral fat mass [18]. However, some studies showed no correlation between mtDNA copy number and regional distribution of adiposity. One study showed no correlation between mtDNA copy number and waist-hip ratio which reflects visceral obesity [30]. Furthermore, results that are opposite to those of our study have also been reported [19, 31, 32]. The investigator of those studies suggested that mtDNA content may increase secondary to mitochondrial dysfunction. Therefore, the association between visceral obesity and mtDNA copy number remains unclear. Our study participants were apparently healthy, young subjects from 20–40 years of age without chronic metabolic diseases. Therefore, although we could not determine causality, our results suggest that visceral fat accumulation may affect mitochondrial DNA content in apparently healthy population without metabolic disturbances.

The precise mechanism that explains the association between mitochondrial DNA copy number and visceral fat mass remains unknown. We could not find the causal factor through our cross-sectional study. However, the results suggest possible mechanisms.

First, increased chronic systemic inflammation according to the secretion of proinflammatory cytokines and adipokines may play important roles in the relationship. Visceral adipose tissue is the main site of secretion of proinflammatory cytokines, which induce mitochondrial dysfunction by affecting signaling pathways associated with mitochondrial biogenesis. Visceral adipose tissue is the main site of secretion of proinflammatory cytokines, which induce mitochondrial dysfunction by affecting signaling pathways associated with mitochondrial biogenesis. For example, in cultured fat and muscle tissue, TNF-*α* depleted endothelial nitric oxide synthase expression along with mitochondrial biogenesis defects and adipocytes with defective TNF-*α* signaling showed partial recovery of mitochondrial function in obese mice [33]. In our study, increased mtDNA copy number was significantly associated with decreased hs-CRP level which reflects the total inflammatory status of human body. Furthermore hs-CRP level was also positively associated with visceral fat accumulation after it was adjusted for age and sex (*r* = 0.5, *P* < 0.05) (data not shown). Because both visceral adiposity and mitochondrial DNA copy number were associated with systemic inflammatory status, increased inflammation level may explain the observed link between visceral obesity and decreased mitochondrial contents. However, the association between visceral fat accumulation and mitochondrial copy number remained statistically significant after adjustment for hs-CRP, suggesting that the association was, at least in part, independent of systemic inflammation. Furthermore, it is impossible to find out the specific roles of proinflammatory cytokines and adipokines in the observed association in our study. Therefore, measurement of proinflammatory cytokines and adipokines should be performed in the future.

Second, free fatty acids that accumulated in the visceral adipose tissue may affect the decreased mtDNA copy number. Increased levels of free fatty acids promote increased synthesis of toxic fatty-acid-delivered metabolites. These metabolites elevate the level of oxidative stress, driving mitochondrial dysfunction [34]. Therefore increased free fatty acids according to the visceral fat accumulation may induce the decreased mitochondrial contents.

This study has several limitations. First, the cross-sectional design of our study cannot determine a causal relationship between mtDNA copy number and visceral obesity and the small sample size is another limitation of the current study. Although the correlation was statistically significant, the low power was another limitation. Therefore, we cannot generalize the results to the population at large. In addition, we did not perform fat biopsy, which is the gold standard for investigation of mitochondrial function. However, it is easier to obtain peripheral blood leukocytes than muscle tissue. And decreased mtDNA copy number in peripheral blood leukocytes correlated well with mitochondrial dysfunction in skeletal muscle [35, 36]. Finally, this study did not measure levels of proinflammatory cytokines and adipokines. Therefore our study cannot directly investigate the role of cytokines and adipokines as mediators between visceral adiposity and reduced mitochondrial DNA copy number. We agree that assessing inflammatory cytokine and adipokine levels will provide additional important information in future studies.

In conclusion, our study shows that peripheral blood mtDNA copy number is associated with abdominal visceral fat area in 94 healthy young-aged subjects. Although the causal direction of the relationship between mtDNA copy number and visceral obesity cannot be determined, our study collectively suggests that decreased mitochondrial contents may be a mediator that links visceral obesity and metabolic disturbances. Further studies are required to better understand the pathophysiological and clinical significance of our findings.

## Figures and Tables

**Table 1:** Clinical characteristics of study subjects (*n* = 94).

Variables	Total (*n* = 94)
Age (years)	29.57 ± 0.95
mtDNA copy number^#^	302.08 (48.98–891.25)
Male (*n*, %)	54 (57.4)
Adiposity index	
BMI (kg/m^2^)	27.70 ± 4.36
Waist (cm)	93.06 ± 11.19
Visceral fat area (cm^2^)	95.22 ± 45.45
Subcutaneous fat area (cm^2^)	245.93 ± 100.14
Blood pressure (mmHg)	
Systolic	125.21 ± 15.59
Diastolic	76.96 ± 12.87
Fasting glucose (mg/dL)	87.77 ± 11.40
Fasting insulin (*μ*IU/mL)^#^	7.51 (4.53–11.84)
HOMA-IR^#^	1.57 (0.97–2.68)
Lipid profile (mg/dL)	
Total cholesterol^#^	181.00 (164.00–215.25)
Triglyceride^#^	89.00 (63.00–131.25)
HDL-cholesterol	52.25 ± 11.92
Hs-CRP^#^ (mg/L)	0.46 (0.10–1.42)
Smoking (*n*, %)	23 (24.5)
Alcohol consumption (*n*, %)	34 (36.2)

Note: BMI: body mass index; HOMA-IR: Homeostasis Model of Assessment of Insulin Resistance; HDL: high-density lipoprotein; LDL: low-density lipoprotein; hsCRP: high sensitive C reactive protein.

Alcohol consumption was defined as drinking alcohol more frequently than once per week.

Normally distributed data are shown as the mean (±SD).

^#^ Non-normally distributed data are presented as medians (25–75 percentiles) and analyzed after log-transformation to correct for skewed distribution.

**Table 2:** The correlation between mtDNA copy numbers^#^ and various parameters.

Variables	Unadjusted		Age, sex adjusted	
	*r*	*P*-value	*r*	*P*-value
Age (years)	−0.28	0.01		
Adiposity index	−0.25	0.01	−0.22	0.04
BMI (kg/m^2^)	−0.33	<0.01	−0.23	0.03
Waist (cm)	−0.40	<0.01	−0.28	0.01
Visceral fat area (cm^2^)^#^	−0.16	0.14	−0.21	0.09
Subcutaneous fat area (cm^2^)				
Blood pressure (mmHg)				
Systolic	−0.22	0.04	−0.11	0.32
Diastolic	−0.29	0.01	−0.15	0.17
Fasting glucose (mg/dL)	0.19	0.09	0.14	0.21
Fasting insulin (*μ*IU/mL)^#^	−0.12	0.23	−0.17	0.12
HOMA-IR^#^	−0.07	0.15	−0.12	0.27
Lipid profile (mg/dL)				
Total cholesterol^#^	−0.09	0.42	−0.03	0.80
Triglyceride^#^	−0.37	<0.01	−0.18	0.09
HDL-cholesterol^#^	0.37	<0.01	0.25	0.02
Hs-CRP (mg/L)	−0.42	<0.01	0.32	0.02

^#^Values analyzed after log-transformation to correct for skewed distribution.

Coefficients (*r*) and *P* values were calculated using the Pearson correlation model.

**Table:** (a) Stepwise model

	*β* coefficient	SE	*P*-value
Visceral fat area	−0.33	0.00	<0.01
Hs-CRP (mg/L)	−0.32	0.05	<0.01
Smoking^#^ (%)	−0.21	0.18	0.01
HDL-cholesterol (mg/dL)	0.21	0.39	0.04

*r* ^2^ = 0.35. Variables included in the stepwise model for mtDNA were age, sex, BMI, alcohol consumption, smoking, systolic BP, total cholesterol, HDL-cholesterol, fasting glucose, HOMA-IR and visceral fat area.

To avoid multi-collinearlity, diastolic BP, triglycerides and subcutaneous fat area were not included in the stepwise model.

^#^Values analyzed after log-transformation to correct for skewed distribution.

**Table:** (b) Non-stepwise model

Variables	*β* coefficient	SE	*P*-value
Age (years)	−0.05	0.02	0.30
Male (%)	0.03	0.20	0.89
BMI (kg/m^2^)	−0.12	0.03	0.43
Smoking (%)	−0.42	0.20	0.03
Systolic BP (mm Hg)	0.00	0.01	0.69
Visceral fat area (cm^2^)	−0.32	0.00	0.03
HDL-cholesterol (mg/dL)	0.21	0.42	0.07
Fasting glucose (mg/dL)	−0.04	0.00	0.66
HOMA-IR	−0.04	0.16	0.41
hs-CRP (mg/L)	−0.19	0.06	0.02

*r* ^2^ = 0.58. Variables included in the non-stepwise model for mtDNA were age, sex, BMI, smoking, systolic BP, HDL-cholesterol, fasting glucose, HOMA-IR and hs-CRP.
